# Self– reported oral health and oral health– related quality of life among patients with diabetes mellitus in a tertiary health facility

**DOI:** 10.1186/s12903-023-03336-w

**Published:** 2024-02-04

**Authors:** Abe Elizabeth Oluwatoyin, Esan Arinola, Oyetola Elijah Olufemi, Adeleye Jokotade

**Affiliations:** 1 Department of Oral Pathology/ Oral Medicine, University College Hospital, Ibadan, Nigeria; 2 Department of Internal Medicine, Endocrinology unit, University College Hospital, Ibadan, Nigeria; 3 Department of Oral Pathology/ Oral Medicine, Obafemi Awolowo University Teaching Hospitals Complex, Ile-Ife, Nigeria

**Keywords:** Diabetes, Oral health, Periodontal diseases, Quality of life

## Abstract

**Background:**

Considering the inter-relationship between Diabetes Mellitus (DM) and oral tissues, assessment of oral health status in relation to glycemic control might be informative about the disease condition, which might be pivotal to appropriate management and ultimately improve life satisfaction. This study therefore aimed to assess the pattern of self-reported diabetes related oral conditions and oral health-related quality of life (OHRQoL) among patients with DM at the University College Hospital (UCH), Ibadan, Nigeria.

**Materials and methods:**

A cross- sectional study using an interviewer administered questionnaire was conducted among in- and out- patients being managed for DM by the Endocrinology unit of the hospital. Data collected included bio-data, medical history, self-reported oral conditions and dental service utilisation. The impact of oral health related quality of life was measured using OHIP-14. Oral examination was done to assess oral mucosal lesions and their oral hygiene status. Data was analysed using SPSS 21.

**Results:**

Eighty-four patients with diabetes consisting 23 (27%) in-patients and 61 (63%) out-patients were enrolled. Males were 26(31%) and females 58(69%); their ages ranged from 22 to 88 years with a mean of 60.9 ± 12.8 years. Most (67%) of the participants had one or more self-reported oral complaints, dryness (20.4%) being the most common and bad breath (4.6%) as the least reported. Poor glycemic level was found to be higher among the in-patients (82%) with random blood glucose > 200 mg/dL during admission, compared to the out-patient participants (51%) who had fasting plasma glucose > 110 mg/dL. Their oral hygiene status was assessed using simplified oral hygiene index and documented as fair (65%), poor (25%) and good (10%). About two-thirds (61%) had ever visited a dentist, majorly due to toothache. Only the pain (21.9%) and physical disability (26.3%) components of OHIP-14 were mainly found to affect the participants’ OHRQOL.

**Conclusions:**

This study found high rate of self- reported DM related oral conditions notably oral dryness, and periodontal diseases particularly as teeth mobility and spontaneous exfoliation. Nonetheless, most of the study participants had fair OHRQoL while its physical pain, functional limitation and psychological disability components were most reported.

## Background

Diabetes mellitus (DM) is a metabolic disorder of chronic hyperglycaemia characterized by disturbances to carbohydrate, protein, and fat metabolism resulting from absolute or relative insulin deficiency with dysfunction in organ systems [1]. The four main types of diabetes are type1 diabetes mellitus (T1DM), type 2 diabetes mellitus (T2DM), other specific types and gestational diabetes mellitus (GDM). This classification was revised by the WHO in 2019 but the former has remained popular. Diabetes mellitus is a well-known chronic metabolic disease which significantly affects patients’ quality of life, and also a substantial risk for morbidity and premature mortality [2]. The global diabetes prevalence in 2019 is estimated to be 9.3% [3], while a meta- analysis by Uloko et al [1] reported a prevalence of 5.77% in Nigeria.

Diabetes mellitus is well known to have multi-systemic complications including central nervous system, cardio- pulmonary, vascular, ocular, renal, dermatological and the immune system. Chronic hyperglycemia which characterizes DM can result in organ complications such as retinopathy, nephropathy, neuropathy, coronary heart disease, peripheral arterial and cerebrovascular disease, cataracts, erectile dysfunction and non-alcoholic fatty liver disease [4, 5]. Nonetheless, oral tissues are not excluded from this array, in that the mouth which is the gateway to the body can serve as the site of initial suspicion of the disease. Possible oral complications of DM include periodontal diseases, tooth loss, peri-implantitis, dental caries, salivary gland hypofunction, sialosis, oral microbiome dysbiosis, candidiasis, taste disturbances, burning mouth syndrome, oral ulcers, delayed wound healing, melanin pigmentation, fissured tongue, benign migratory glossitis (geographic tongue), temporomandibular disorders, and osteonecrosis of the jaw [6–10]. These complications are the sequelae of chronic hyperglycemia and could be a pointer to screening for DM in undiagnosed patients, and as well serve as clinical indices for predicting glucose control in patients with DM [11–13].

The World Health Organization Quality of Life (WHOQOL) group defines quality of life as individuals’ perceptions of their position in life in the context of culture and value systems in which they live, and in relation to their goals, expectations, standards and concerns [14, 15]. Oral health problems have a significant impact on an individual’s physical, social and psychological well-being. They can also cause damage that can lead to a self-awareness disorder, pain, discomfort, functional limitation and dissatisfaction with their appearance thereby causing physical, psychological and social limitations [15]. Considering the interaction between diabetes and oral tissues, the assessment of oral health related quality of life (OHRQL) in diabetic patients is pivotal to enhance their management and ultimately improve their satisfaction with life [14]. People diagnosed with DM are at risk of developing oral diseases as a result of being untreated or poorly treated; or due to the side effects of glucose- lowering medications which can affect their health- related quality of life. According to study reports, there are variations to the effect of DM on patients’ OHRQoL. While some documented its impact as being significant, others claimed that DM had little or no impact on OHRQoL among their study population. Two Iranian studies conducted among patients with DM revealed that though OHRQOL was not adversely affected, only the psychological and functional disability components were noted with low OHRQOL [14, 16]. On the contrary, a systematic review by Cervino et al [5] revealed significantly reduced OHRQOL among patients with DM, being related to either local or systemic complications of the disease. Over a decade ago, Oyapero et al. [17] reported that oral health status has impact on the quality of life among patients with DM in a teaching hospital at Lagos Nigeria. However, there has been no study which related the oral health status of DM patients with their OHRQOL at the health facility where this study was conducted. In view of assessing the bi-directional relationship between oral and systemic diseases to predict if oral lesions could serve as clinical indicators for disease severity, our research hypothesis was to assess the variation of oral health status between in- and out-patients in patients with DM, as well as its impact on their oral health and overall health. This study aimed to determine patients’ awareness of oral complications of their diabetic condition through self- reporting. The participants’ reports were evaluated alongside oral examination findings during the study including pattern of oro-mucosal lesions, oral hygiene status and practices.

## Methodology

The study was conducted among in- and out- patients being managed for DM by the Endocrinology unit at University College Hospital, Ibadan, Nigeria. Ethical approval was obtained from the University of Ibadan and University College Hospital, Ibadan Joint Ethical Review Committee. The study participants included patients with diabetes who were either newly diagnosed or already on medications seen at the medical out-patient clinic, as well as those admitted on the wards for in-patient care. Patients who had DM-related medical emergencies were excluded.

An interviewer-administered questionnaire method was used for data collection which included age, gender, marital status, level of education and religion. Questions related to their DM status like duration of diagnosis, co-morbidities, anti-diabetes medications, blood glucose profile including random blood glucose (RBG), fasting plasma glucose (FPG) and glycated hemoglobin (HbA1c) were documented. Further questions on their past experiences of diabetes- related oro-facial conditions, oral hygiene practices and dental service utilization were also documented. Oral examination was carried out by an oral physician under natural light using disposable wooden spatula, latex gloves and face mask. Oral hygiene status was assessed using the Simplified Oral Hygiene Index (OHI-S) [18] and documented as either poor (0-1.2), fair (1.3-3.0) or good (3.1-6.0).

The oral health-related quality of life (OHRQoL) was assessed using Oral Health Impact Profile (OHIP – 14) which tested seven composite domains namely; functional limitations, physical pain, psychological discomfort, physical disability, psychological disability, social disability and handicap. Each question from OHIP-14 was assessed using a 5-point Likert scale indicating “always” (4), “fairly often” (3), “occasionally” (2), “hardly ever” (1), or “never” (code 0). The sum of responses for each question was added to generate an overall OHIP-14 score, with possible scores ranging from 0 to 56. The sum OHIP score of 14 or less is indicative of low impact while score of 15 or more is indicative of high impact, similarly adopted by Isiekwe et al [19].

Data was analysed using SPSS version 25. Continuous variables like age, duration of diagnosis and glycated hemoglobin level were summarized using means and standard deviation. Qualitative variables such as gender, medication types and self-reported oro-facial lesions were expressed as proportions and percentages. Pearson´s Chi-square test was used to assess the association between the independent variables and the outcome variables using significance level of 5% (p < 0.05).

## Results

A total of 84 patients with DM consisting 23 (27%) in-patients and 61 (63%) out-patients were assessed for the study; having a gender distribution of 26(31%) males and 58(69%) females while their ages ranged from 22 to 88 years with a mean of 60.9 ± 12.8 years. More than half (51.2%) of the study participants were elderly patients of sixth decade of life and beyond, followed by 44.0% who were in the fourth and fifth decades of life. (Table 1)

**Table 1:** Socio-demographic features of the study participants

Category	Frequency (N)	Percentage (%)
**Gender**		
Male	26	31
Female	58	69
**Age range**		
21–40 years	4	4.8
41–60 years	37	44.0
> 60 years	43	51.2
**Patient type**		
In-patient	23	27
Out-patient	61	63
**Marital status**		
Single	5	6
Married	57	68
Widow	22	26
**Educational status**		
Nil	11	13
Primary	21	25
Secondary	25	30
Tertiary	27	32

### Assessment of diabetes status among the study participants

Almost (95%) all the study participants had been diagnosed with Type2 DM, 4% had Type1 DM while a few (1%) were unspecified. Majority (58%) had been diagnosed to have diabetes mellitus for < 10 years, only a few (6.2%) had been diagnosed for over 20years. About one-third (33.3%) of the participants are being diagnosed with Hypertension alongside DM. Various anti-DM regimen being used by the participants include Insulin, Metformin, Sulfonylurea, Dipeptidyl peptidase 4 (DPP4) inhibitors and combination regimen. (Table 2)

Concerning the assessment of glycaemic control among the participants, most (82%) of those who were in-patients had random blood glucose > 200 mg/dL on admission. On the other hand, about half of the out-patients had FPG ≤ 110 mg/dL. Only two-thirds of the study participants had HbA1C recorded; the mean (± SD) values were higher among in-patients (11.5 ± 2.9) compared to out- patient (8.4 ± 3.1) participants.

**Table 2:** Assessment of diabetic status and drug regimen among the study participants

Diabetes assessment	Frequency (N)	Percentage (%)
**Diabetes type**		
Type 1	3	3.6
Type 2	80	95.2
Unspecified	1	1.2
**Year(s) of diagnosis**		
< 10 years	47	58
11–20 years 21 years and above	29 5	35.8 6.2
**Co-morbidity/ Systemic DM**		
**complications**	28	33.3
Hypertension	12	14.3
DM-foot ulcer	6	7.1
Kidney disease	6	7.1
Hyperosmolar hyperglycemic state	2	2.4
Erectile dysfunction	5	6.0
Cellulitis	1	1.2
Ocular dysfunction	1	1.2
Thyroid dysfunction	3	3.6
Retroviral disease	20	23.8
Nil		
**Anti- diabetes regimen**		
Insulin alone	15	18.5
Insulin + oral agents	22	27.2
Metformin alone	17	21.0
Metformin + Sulfonylurea	25	30.9
Metformin + DPP4 inhibitors	2	2.5

**Fig. 1:**
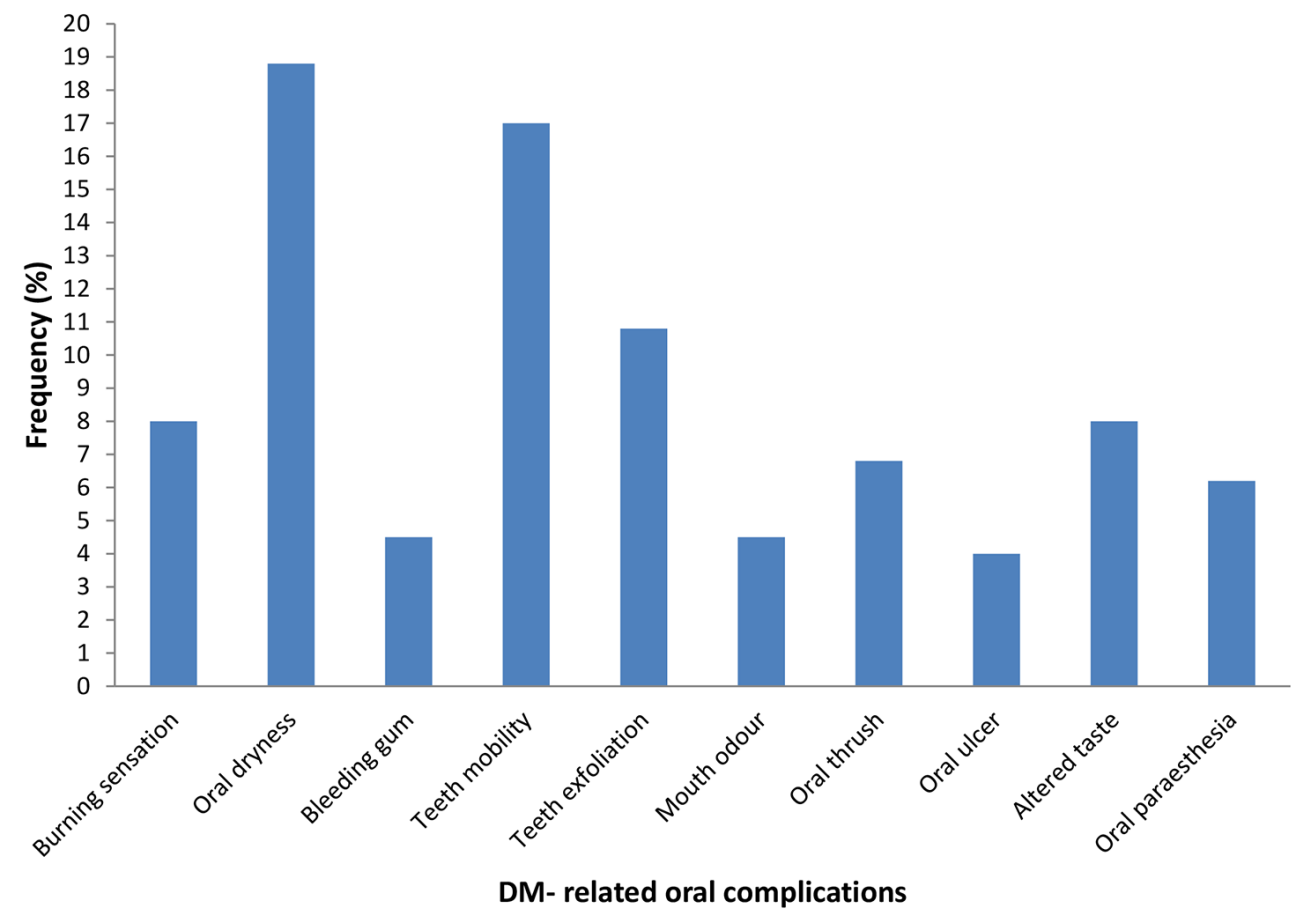
Self- reported Diabetes- related oral complications among the study participants

### Self- reported oral conditions associated with diabetes mellitus

Overall, two-thirds (67%) of the participants had one or more oral complaints. Oral dryness (xerostomia) was the most (19.3%) self-reported oral condition followed by teeth mobility (17.4%), spontaneous tooth exfoliation (10.6%), burning sensation (7.5%), altered taste (6.8%), oral paraesthesia (6.8%) and mouth odour (4.3%) as the least reported. (Fig. 1)

Comparing the participants’ sub-groups, more participants among the out-patients (78.6%) indicated to have experienced DM-related oral conditions than the in-patients (21.4%) (p = 0.08).Though not statistically significant, majority of the participants with poor glycemic control (76.5%) were noted having self-reported complaints compared with those having good glycemic control (23.5%) (p = 0.83).

### Oral examination findings among the study participants

**Fig. 2:**
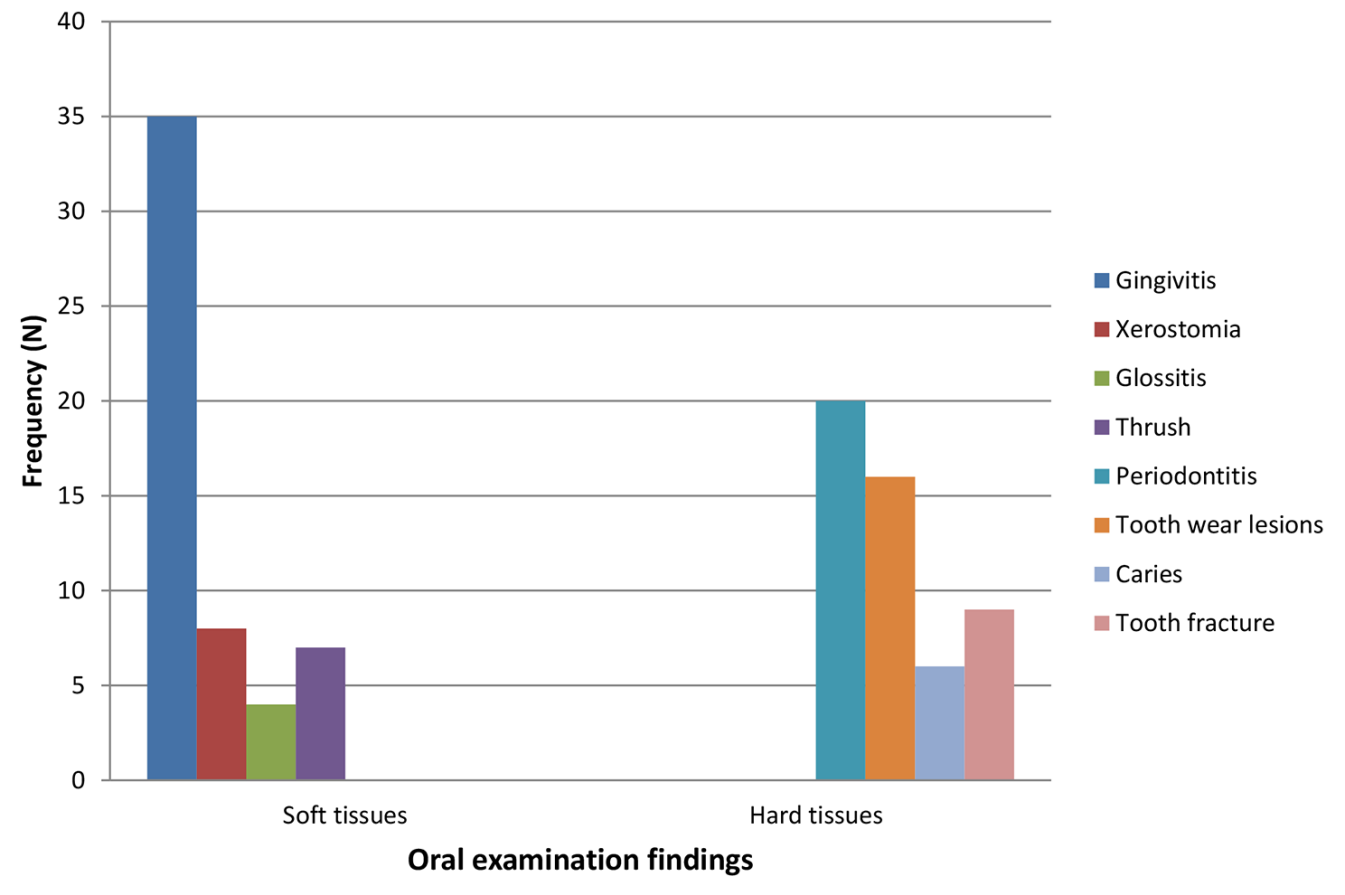
Pattern of Oral lesions seen among the study participants

Oro-facial examination conducted on the study participants revealed varying patterns from soft to hard tissue- related pathologies. Notably, periodontal diseases (gingivitis, chronic periodontitis, periodontal abscess, tooth loss due to periodontitis) were seen both as soft and hard tissue pathologies. (Fig. 2) Other oral diseases seen were caries, tooth wear lesions, fractured teeth, xerostomia, glossitis and oral thrush. Majority had combination of soft and hard tissue lesions (46.4%), 23.8% had soft tissue lesions alone and 14.3% having just hard tissue lesions. Only 15.5% did not have either soft or hard tissue lesions.

### Oral hygiene practices and dental service utilisation

More than half (58%) of the participants clean their teeth using tooth brush and toothpaste only, while 35% of them use chewing stick alongside toothbrush and toothpaste. A minority (5%) use chewing stick only for their oral care. Similarly, majority (70%) of the participants clean their teeth once daily, while the remaining (30%) clean twice or more. Oral examination done among the study participants revealed their oral hygiene status mainly as fair (65%), followed by poor (25%) and good (10%) status. Most of those with poor oral hygiene observed once daily brushing method (70%) as well as teeth cleaning with chewing stick only (60%).

About two-thirds (61%) of the study participants noted to have ever visited a dentist, majorly for the complaint of toothache, and other reasons like mouth odour, dental cleaning and for routine oral check-up. The most commonly reported treatment done was extraction, followed by scaling and polishing, dental filling and denture fabrication. The remaining one-third who had never visited a dentist mostly reported that they had no dental complaint for which they could have sought a dental consultation.

### Oral health-related quality of life (OHRQoL) assessment using OHIP – 14

The mean score of OHIP for all participants was 1.18 (± 0.38). Most (82%) of the participants had total OHIP score of 14 or less. Nonetheless, using each domain component, physical pain, functional limitation and psychological disability components of OHIP-14 were found to affect majority of the participants’ OHRQOL. (Table 3)

**Table 3:** Distribution of the participants’ responses to questions on OHIP-14

Questions	Never (%)	Hardly ever (%)	Occasionally (%)	Fairly often (%)	Always (%)
**Functional limitation**					
1. Trouble pronouncing any word	75 (90.4)	0 (0)	8 (9.6)	0 (0)	0 (0)
2. Felt that your sense of taste has worsened	63 (76.8)	2 (2.5)	16 (19.5)	1 (1.2)	0 (0)
**Physical pain**					
3. Had painful ache in your mouth	64 (79.0)	1 (1.2)	12 (14.8)	3 (3.8)	1 (1.2)
4. Found it uncomfortable to eat any food	56 (67.5)	1 (1.2)	24 (28.9)	2 (2.4)	0 (0)
**Psychological discomfort**					
5. Being self-conscious	60 (72.3)	1 (1.2)	6 (7.2)	2 (2.4)	14 (16.9)
6. Felt tense/ nervous	69 (84.1)	3 (3.7)	9 (11.0)	0 (0)	1 (1.2)
**Physical disability**					
7. Diet been unsatisfactory	60 (72.3)	1 (1.2)	20 (24.1)	2 (2.4)	0 (0)
8. Had to interrupt your meals	55 (67.9)	2 (2.5)	23 (28.4)	0 (0)	1 (1.2)
**Psychological disability**					
. Found it difficult to relax	64 (78.0)	3 (3.7)	13 (15.9)	0 (0)	2 (2.4)
10. Felt a bit embarrassed	75 (94.9)	1 (1.3)	3 (3.8)	0 (0)	0 (0)
**Social disability**					
11. Felt a bit irritable with other people	69 (86.3)	3 (3.8)	7 (8.6)	1 (1.3)	0 (0.0)
12. Had difficulty doing your usual jobs	63 (75.9)	2 (2.4)	15 (18.1)	1 (1.2)	2 (2.4)
**Handicap**					
13. Felt that life in general was less satisfying	70 (88.6)	1 (1.3)	6 (7.6)	0 (0)	2 (2.5)
14. Being totally unable to function	70 (87.5)	1 (1.3)	7 (8.8)	0 (0)	2 (2.5)

## Discussion

Diabetes mellitus as a chronic metabolic disease exerts its deleterious effects on most human systemic components especially if uncontrolled or not appropriately managed. The findings from this study confirmed the multi- dimensional complications associated with DM including oro-facial tissues. Most of our study participants reported one or more oral conditions in relation to their DM status. Oral dryness (xerostomia) was the most self-reported oral condition which has been significantly reported among patients with DM [2, 13, 20–22]. We found poor glycemic control among a greater proportion of the participants as evidenced by increased level of glycated hemoglobin. Oral dryness could possibly result from polyuria due to chronic hyperglycemia, poly-pharmacy/ medication side effects, microvascular changes and alterations in the basement membranes of salivary glands [23]. Co-morbid conditions like chronic kidney disease may be contributory to oral dryness [2, 24]. Oral dryness can present with problems such as difficulty in eating, swallowing, and speaking, consequential to negative effect on patients’ quality of life [23].

Halitosis may feature as a complication of uncontrolled DM especially in Type 1DM, manifesting as ketotic breath. While halitosis (bad breath) was the least self –reported oral condition in this study, some authors [13, 20]reported halitosis as a significantly common finding in their studies especially among uncontrolled DM patients. This study further revealed periodontal diseases as part of self-reported symptoms notably gum bleeding, teeth mobility and sponteous exfoliation; which have been profoundly documented in the literature [2, 8, 17]. The interplay between poorly controlled DM and periodontal disease has been significantly reiterated from various documentations [8, 25–27]. There is strong evidence that diabetes is a risk factor for gingivitis and periodontitis, and the level of glycemic control appears to be an important determinant in this relationship. The systemic effects of diabetes contribute to periodontal disease through accumulation of advanced glycation end products, increased pro-inflammatory cytokines, increased oxidative stress and impaired host defense mechanism. Studies have shown how chronic hyperglycemia produces advanced glycation end products (AGEs) that can bind to specific receptors cells such as fibroblast, endothelial cells and macrophages, increased pro-inflammatory cytokines, vascular modifications, altered healing and increased predisposition to infections [24, 27].

Diabetes related oral health problems affect the quality of life of patients leading to physical inactivity and functional limitation. One of such is burning sensation (dysesthesia) which has been linked to poor glycemic control, metabolic alterations in oral mucosa, microvascular angiopathy, candida infection, and neuropathy [4, 29, 30]. Few participants in this study reported to have experienced oral burning sensations since DM diagnosis as similarly documented by Madathil et al. [31]. Considerably, this neuropathic condition may affect physical and psychological functions, sleep disturbance, anxiety, and depression [23]. Some authors documented a negative impact of DM on OHRQoL especially among uncontrolled DM patients [32, 33]. In a study by Azogui-Lévy et al., poor oral health was linked to negative impact on OHRQoL in patients with diabetes, alongside pain and discomfort on mastication [34]. However, this study found that oral complications of diabetes did not adversely affect OHRQoL, being similarly documented by Allen et al. [35] and Sadeghi et al. [14]. Nonetheless, this study approves that dentists and physicians play an important role in improving diabetic patients’ knowledge regarding oral complications and their effect on patients’ quality of life.

## Conclusion

Majority of the study participants had poor glycemic control which was in tandem with increased self- reported oral conditions and oro-mucosal lesions found on oral examination. While most of our study participants had fair oral hygiene status and dental service utilisation, routine oral care in DM patients should be prioritised for improved OHRQOL. Although oral health-related QoL appeared not to be adversely affected by their DM status, it is imperative to prioritize as well as emphasize the importance of oral health as a vital component of holistic care for patients with DM through dynamic Dentists - Physicians collaboration.

## Data Availability

All data generated or analysed during this study are included in this published article.
